# The letter as a forum to promote diversity in dementia research

**DOI:** 10.1590/1980-5764-DN-2023-0029

**Published:** 2023-06-05

**Authors:** Timothy Daly

**Affiliations:** 1 Flacso Argentina, Bioethics Program, Buenos Aires, Argentina.; 2 Sorbonne Université, Science Norms Democracy UMR 8011, Paris, France.

Dear Editor,

Three major obstacles for dementia researchers in Latin America have been identified: funding constraints, language barriers, and article processing charges (APCs)^1^ ^,^ ^2^. It is vital that structural changes are made to facilitate access to publication in low- and middle-income countries (LMICs)^1^. But while waiting for such changes, I highlight a complementary initiative that LMIC researchers, particularly early-career researchers (ECRs), may benefit from on how to publish their ideas and empirical research: the writing of *Letters to the Editor* in dementia journals such as *Dementia & Neuropsychologia*.

Letters can overcome all three aforementioned barriers (funding, language, APCs). They are short-format articles typically between 250 and 750 words in response to recent publications, relevant topics, or research letters showcasing empirical research. Letters are generally free to publish; many fully open-access journals do not levy APCs for authors of letters, but always check submission guidelines for suitability and APCs before sending to any journal. In such cases, it costs little time and no money to write and publish a letter. Due to its brevity, a letter is a reasonable first publication for trainee researchers whose first language is not English^2^, and can be a vital source of motivation and validation during the difficult ECR period^3^. Furthermore, ECRs are usually already engaged in journal clubs, which they might use to write group or individual letters or, otherwise, build on their independent reading of the literature^3^. 

*Dementia & Neuropsychologia* provides examples of recent letters showcasing empirical work^4^, responses to recent publications^5^, and general topics^6^. While the history of contemporary dementia research suggests that short pieces can have significant impact^7^, LMIC ECRs should not become over-reliant on letters as a replacement for longer pieces^2^. 

Thus, the letter is a tool to help LMIC ECRs gain visibility in dementia research while awaiting necessary structural changes for academic publication. Suggestions for writing a good letter can be found elsewhere^8^.
